# Editorial: Preventing sarcopenia and promoting musculoskeletal health in middle-aged adults: the role of exercise and nutrition

**DOI:** 10.3389/fspor.2025.1601326

**Published:** 2025-04-11

**Authors:** Theocharis Ispoglou, Catherine Norton, Deaglan McCullough

**Affiliations:** ^1^ Carnegie School of Sport, Leeds Beckett University, Leeds, United Kingdom; ^2^ Department of Physical Education & Sport Sciences, University of Limerick, Limerick, Ireland

**Keywords:** sarcopenia, resistance training, ultra-processed foods, gut-muscle axis, middle-aged adults, muscle protein synthesis, anabolic resistance, gender-specific barriers

**Editorial on the Research Topic**
Preventing sarcopenia and promoting musculoskeletal health in middle-aged adults: the role of exercise and nutrition

Sarcopenia, once considered an inevitable consequence of ageing, is now recognised as a complex syndrome influenced by lifestyle, disease, and acute physiological stress. As global life expectancy rises, its prevalence is increasing, placing a burden on healthcare systems (1, 2) due to its association with disability, frailty, and comorbidities (3). Prevalence estimates range from 0.2% to 86.5% depending on diagnostic criteria (4). While typically studied in older adults, evidence suggests an earlier onset, with rates between 8% and 36% in those younger than 60 years and between 10% and 27% in those aged 60 years and older (4). This variability partly reflects differences in classification, with the European Working Group on Sarcopenia in Older People (EWGSOP2) (5) defining primary sarcopenia (driven by ageing) and secondary sarcopenia (driven by disease, inactivity, or malnutrition), each of which poses distinct diagnostic challenges.

Acute sarcopenia, which refers to rapid muscle loss following illness or hospitalisation, has gained attention but remains inconsistently defined. Before Welch et al. (6) introduced the term “acute sarcopenia”, this phenomenon was typically defined as disease-related muscle wasting. EWGSOP2 (2019) (5) incorporated acute sarcopenia in its diagnostic framework, but its clinical application remains limited. A recent systematic review by Aldrich et al. (7) found that acute sarcopenia develops in 18% of hospitalised patients, reaching 59% in intensive care settings; however, current diagnostic criteria may underestimate muscle deterioration, impacting recovery, quality of life, and long-term health outcomes.

Despite ongoing efforts by multiple working groups (8), variability in definitions and diagnostic criteria hinders clinical recognition and intervention. Strengthening collaboration between organisations such as EWGSOP2 and the Global Leadership Initiative on Sarcopenia remains critical to advancing standardised diagnostic frameworks.

## Early intervention: a critical window for prevention

While diagnostic precision is important, prevention must not be delayed, particularly in middle-aged adults, where early intervention can prevent muscle loss. Midlife is a key period for musculoskeletal health, with declines in neuromuscular efficiency (9), physical activity (10), hormonal alterations (e.g., oestrogen) (11), and poor dietary habits accelerating muscle loss (12, 13). Gender-specific barriers also influence the risk of sarcopenia, particularly in women during the perimenopausal phase (14, 15). Developing inclusive, accessible resistance training (RT) programmes tailored to women's needs – addressing social, cultural, and practical barriers – could improve long-term engagement.

**Figure 1:**
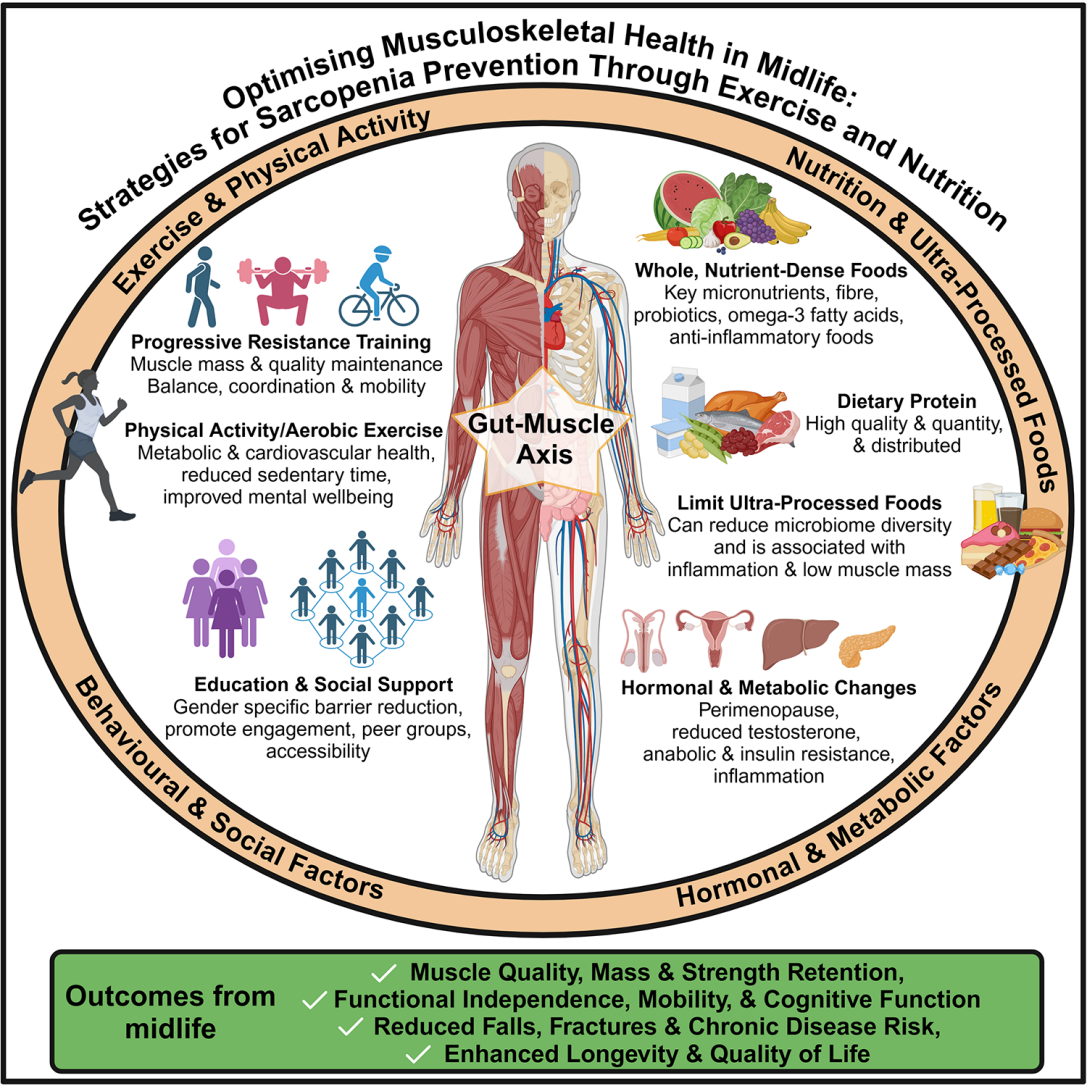
Lifestyle and behavioural strategies—including exercise, nutrition, and social factors—to support musculoskeletal health and prevent sarcopenia in midlife.

A summary of the key intervention strategies and modifying factors for the prevention of sarcopenia in midlife is presented in Figure 1. This Research Topic explored key lifestyle approaches, including nutrition, exercise, and gut-muscle interactions, offering solutions for sustaining musculoskeletal health through early, targeted intervention.

## Ultra-processed foods (UPFs) and muscle deterioration

Diet is fundamental to muscle health, but modern dietary patterns are increasingly dominated by UPFs, which may accelerate muscle loss. Kong et al. investigated the association between ultra-processed food (UPF) consumption and muscle health, revealing that higher UPF intake was significantly associated with low muscle mass in young to middle-aged adults. These findings reinforce the need to prioritise whole, nutrient-dense foods to reduce early muscle deterioration.

However, not all UPFs are inherently harmful. High-protein products, such as protein shakes and bars, fall into the UPF category but can support protein intake when consumed as part of a balanced diet. Alternatives, such as high-protein yoghurts containing probiotics, may also promote gut health. Future research should assess diet quality holistically, considering both benefits and risks when evaluating UPFs in muscle health strategies.

### The gut-muscle axis: a novel target for sarcopenia prevention

Emerging research highlights the gut-muscle axis as a key regulator of muscle health, linking gut microbiota to metabolism, inflammation, and function. Microbiome-targeted interventions may complement exercise and nutrition, offering therapeutic potential beyond sarcopenia prevention (16).

Li et al. examined exercise-induced microbiome shifts and suggested that they might enhance muscle protein synthesis and metabolic resilience. Furthermore, diet critically influences gut health, with UPFs [associated with low muscle mass (Li et al.)] shown to reduce microbial α-diversity and increase proinflammatory bacteria (17, 18). By linking gut health to muscle function, new holistic health strategies may help mitigate muscle loss, metabolic disease, autoimmune disease, and chronic inflammation. While promising, causal mechanisms remain unclear, and future research must determine whether microbiome modulation – through diet, probiotics, or exercise – can offer viable therapeutic approaches for sarcopenia and broader health outcomes.

## The protein paradox: why diet alone is insufficient

While protein remains crucial, diet alone is insufficient without exercise. Research shows that muscle protein synthesis declines with age, necessitating higher protein intake thresholds to stimulate an adequate anabolic response. Reviews highlight protein quality, timing, and distribution as key factors in maximising muscle preservation (19, 20).

Schalla et al. found that high-protein diets had minimal impact on muscle mass and strength in physically active middle-aged adults, reinforcing the need for an integrated approach combining diet and RT. This highlights a gap in current nutritional recommendations, where protein supplementation is often prioritised over exercise (i.e., RT), limiting its efficacy in the prevention of sarcopenia.

## Exercise as a primary prevention strategy

Of all interventions, exercise (i.e., RT) remains the most effective strategy for preventing muscle loss and function. Li et al. conducted a network meta-analysis that identified RT as the most effective approach to counteracting sarcopenia, particularly in clinical populations.

Despite strong evidence supporting RT, participation remains low, with global participation rates of 18%–35% in men and 14%–26% in women (14). Common barriers, especially among midlife women, include perceived time constraints, lack of knowledge and education, exercise modality preferences, and social factors (15). Addressing these barriers through education, community-based programmes, and gender-inclusive training environments is crucial to improving adherence and maximising public health benefits.

## Conclusion

Despite the growing recognition of sarcopenia as a midlife health concern, prevention efforts remain fragmented. This collection highlights the urgent need for integrated, proactive strategies targeting modifiable lifestyle factors – diet, resistance exercise, and gut health – before significant muscle loss occurs.

Public health initiatives must prioritise early intervention with a coordinated effort to educate healthcare professionals, policymakers, and the public on the importance of exercise and nutrition for muscle health. While resistance exercise remains the most effective intervention, complementary nutritional strategies should focus on reducing UPF consumption, promoting gut health, and ensuring adequate, high-quality protein intake to maintain muscle.

Policymakers and healthcare systems must invest in accessible, evidence-based interventions tailored to midlife adults, embedding sarcopenia prevention within public health frameworks. Future research should enhance the accessibility, adherence, and long-term sustainability of interventions, making sarcopenia prevention a midlife priority rather than an afterthought.
